# Perspectives of farmers and tourists on agricultural abandonment in east Lesvos, Greece

**DOI:** 10.1007/s10113-017-1276-4

**Published:** 2018-02-02

**Authors:** Cecilia Zagaria, Catharina J. E. Schulp, Thanasis Kizos, Peter H. Verburg

**Affiliations:** 1 Environmental Geography Group, Institute for Environmental Studies, Vrije Universiteit Amsterdam, De Boelelaan 1087, 1081 HV Amsterdam, the Netherlands; 2 Department of Geography, University of the Aegean, Mytilene, Greece; 3 Swiss Federal Research Institute WSL, Research Unit Landscape Dynamics, Zürcherstrasse 111, 8903 Birmensdorf, Switzerland

**Keywords:** Farmer typology, Landscape change, Traditional farming, Landscape preference, Olive plantations

## Abstract

Multi-stakeholder perceptions of landscape changes are increasingly recognized as essential inputs to discussions on future landscape developments, particularly when addressing the future of European rural areas experiencing agricultural abandonment. This research presents a case exploration of abandonment of olive plantations in east Lesvos, Greece. We conducted two sets of semi-structured interviews to relate an exploration on local farmers’ ability and willingness to maintain the plantations, to the results of a landscape preference survey undertaken with tourists. Three farmer types are identified following a cluster analysis based on attributes of individual ability and willingness to farm. Farmers belonging to the prevalent type revealed low ability and willingness and expect to further extensify their farms. The remaining two farmer types have higher willingness; they are motivated by cultural reasons, more frequently expressing a desire to maintain their land under family ownership, and partake in social cooperative initiatives promoting practices valorizing the olive plantations. We outline how these types interact with regional drivers of change, and partly also contribute to persistence of abandonment through constrained ability to farm. Abandonment does not align with current landscape preferences of tourists, who favor cultivated landscapes, elements of traditionality within built infrastructure and undertake nature-based activities. We discuss how high willingness to farm associated with professional and pluri-active forms of farming may however provide opportunities to maintain the cultivated landscape and synergize with (agri-)tourism demand. Our findings are comparable to those of other European studies, contributing to discussions on the future of its rural landscapes.

**Electronic supplementary material:**

The online version of this article (10.1007/s10113-017-1276-4) contains supplementary material, which is available to authorized users.

## Introduction

European agricultural landscapes are increasingly defined as multifunctional, recognized for their multiple roles in producing materials, conserving the environment and sustaining rural vitality (Kurkalova 2005; Wilson 2008; Van Zanten et al. 2014). The European Landscape Convention, adopted in 2000, played a role in formalizing and promoting this recognition by calling for an integrated framing of landscape assessment and management, where the landscape is defined as a material manifestation of complex human-environment dynamics in a given place, as perceived by a given observer (Council of Europe 2000; Pinto-Correia and Kristensen 2013). This approach has been implemented in regulatory policy as well as in more persuasive and educational measures (Primdahl et al. 2013). Implementations have acknowledged the role of both landscape manager and user in shaping the landscape, changing the physical environment and public perceptions of “rurality” (Fyhri et al. 2009; Sayadi et al. 2009).

Acknowledgement of the multiple services provided by agricultural landscapes mirrored the emergence of novel agricultural transitions diverging form solely productivist landscapes. Agricultural re-structuring towards non-commodity land outputs has been seen, for example, in the utilization of agricultural spaces for leisure (Buijs et al. 2006; Oliver and Jenkins 2003). Wilson (2008) differentiates between cases of “weak” to “strong” multifunctionality, where, under strong multifunctionality, societal values, demands and agri-environmental functioning align. He hypothesizes extensively farmed upland areas of high conservation value within developed countries have high potential for strong multifunctionality, partly due to higher frequency of pluri-active farmers engaged in supporting tourism and landscape protection.

Agricultural restructuring in upland farming areas, however, has often resulted in marginalized territories witnessing economic decline and public disinvestment (Rizzo 2016). European projections forecast agricultural abandonment as a dominant land use change trajectory in the forthcoming 20 years, particularly affecting mountainous, remote regions characterized by extensive, smallholder systems (van der Zanden et al. 2017). This process imposes important, context-dependent, ecological and societal trade-offs (Munroe et al. 2013; Renwick et al. 2013) which include implications for biodiversity, carbon storage (Plieninger et al. 2014; Stürck et al. 2015), recreation, cultural heritage, forest fire vulnerability, and soil and water resources (Beaufoy 2001; Sayadi et al. 2009; van der Zanden et al. 2017). As a result, a policy debate has emerged on how to best manage this transition, favoring the preservation of the cultivated landscape or the support of rewilding processes. This decision is inevitably rooted (and complicated) by the different, dynamic perceptions and values attributed by people to the landscapes in question (Navarro and Pereira 2015).

The former municipality of Gera (87 km^2^), situated in east Lesvos (Greece), presents a mountainous rural region experiencing abandonment. Gera’s olive-dominated landscape is changing towards a Mediterranean-type forest, averaging land conversion rates of 34 ha/year over the last 50 years, while experiencing rural depopulation (Bürgi et al. 2015; Kizos and Koulouri 2010). The olive landscape has been defined as traditional due to the widespread and longstanding presence of the plantations, remnants of heritage elements including dry stonewalls and terraces within smallholder systems, and prevalence of manual labor over mechanization (Kizos and Koulouri 2006).

Extensive research has taken place in the region to identify the following: (1) regional drivers of landscape change (Kizos and Koulouri 2006, 2010; Kizos and Spilanis 2008), (2) heterogeneous groups of olive-producing farmers and farm types (Giourga et al. 2008; Kizos et al. 2010; Kizos and Koulouri 2010), (3) different management practices altering landscape features on the farm (Kizos et al. 2010; Kizos and Koulouri 2006, 2010), and (4) how changing features at the farm scale result in aggregate processes of transformation in the overall landscape (Kizos et al. 2010; Kizos and Koulouri 2006; Kizos and Spilanis 2008). These can be interpreted as four steps in a sequenced exploration of regional landscape change through the analysis of farm-scale dynamics. While insight has advanced in each of these focus areas, linking processes between steps (1), (2), and (3), i.e., how actors are influenced by regional drivers to undertake specific actions on the landscape, demand additional research. Importantly, previous studies revealed a farmer classification based on household dependency on agricultural incomes alone is a poor predictor for different managerial strategies, and thus observable landscape changes. Different farmer decision-models are needed, able to capture the role of place attachments held by farmers to the cultivated landscape and accurately weigh the influence of regional drivers upon faming dynamics (Kizos et al. 2010).

These novel decision-making models may reveal whether farmers are able and/or willing to maintain the cultivated landscapes, and in which conditions. A question which arises is how these landscape changes may be perceived, and in-turn impacted by, non-farmer landscape users. Lesvos has limited social and economic development opportunities beyond tourism, yet this sector remains underdeveloped, not having witnessed the mass-tourism character of other Greek destinations (Giourga et al. 2008; Loumou et al. 2000). The “tourism centers” of the island have been associated with more stable population numbers and lower rates of abandonment (Loumou et al. 2000). Studies report this occurs as tourism provides a means of both on- and off-farm income diversification for farmers. The importance of complementary off-farm employment is notable in the number of pluri-active farmers in the region. The latest population census (2011) identified a discrepancy between the 350 individuals (21% of Gera’s population) with primary occupation in agriculture and 1538 active farms. On-farm income diversification through agri-tourism has been promoted by regional authorities. Despite successfully increasing incomes, agri-tourism has failed to truly integrate activities within the cultivated landscape and associated traditional products (Gousiou et al. 2001). An influence of tourism on landscape composition has been suggested, yet there has been no clear assessment of demand by this user group for cultivated landscapes in particular, especially uncertain as greatest influence is being witnessed not inland but rather in proximity of coastal centers.

Discussions on interventions addressing the future of European rural landscapes experiencing abandonment demand an understanding of the multifunctional potential of these spaces, partly determined by the demands and perspectives of affected landscape makers and users. Through this case study, we aim to identify if and how olive farmers in east Lesvos are able and willing to maintain the cultivated landscape, and discuss how the landscape changes which ensue from their actions relate to landscape preferences of tourists. Following a presentation of conceptual and methodological backgrounds, the paper is structured around its three objectives: (1) to construct a farmer typology based on individual attributes outlining ability and willingness to farm, characterizing the decision-making behavior of local farmers; (2) to explore how the identified farmer types undertake actions on the landscape with implications for abandonment or maintenance of the cultivated landscape; and (3) to provide a preliminary investigation on the landscape preferences of tourists, relative to the landscape change trajectories identified in east Lesvos.

### Conceptualizing landscape change in east Lesvos

#### Landscapes of change

We differentiate between landscape changes occurring at the farm-scale to changes at the regional scale caused by the cumulative influence of farmer actions. Farmer actions can be clustered in four separate groups: intensification (increasing inputs, frequency of management), diversification (construction of agri-tourism infrastructure, switch to mixed cultivation), expansion, or disinvestment (extensification, sale, abandonment). These actions are reflected in the primary change trajectories identified within olive-dominated plantations in east Lesvos: abandonment, diversification and agricultural intensification (Kizos and Koulouri 2010) (see Online Resource 1 for a comprehensive description of Lesvos-based/Mediterranean olive plantation typologies identified in literature, and related change trajectories).

#### Drivers of change

Abandonment is partly driven by declining *olive oil prices* and a difficulty in intensifying mountainous (poorly accessible) smallholder olive plantations (Bürgi et al. 2015). This has contributed to a declining perception of farming as a desirable profession for younger generations. Farmers are also heavily reliant on *agricultural subsidies*; however, age and agricultural income dependency requirements for some subsidies exclude a portion of retired and pluri-active farmers (Kizos and Spilanis 2008). *Demand for (agri-)tourism services* has resulted in some plantation clearing for agri-tourism development and housing pressures particularly near coastal centers (Kizos and Koulouri 2010). It furthermore provides opportunities for off- and on-farm income diversification to farmers. Declining levels of social capital also play a role, as “traditional” farmer *cooperatives* with political affiliations are distrusted. Novel, “social” cooperative forms are emerging, centered on promoting cultural values associated with the landscape to boost profitability and employment (Bürgi et al. 2015; Shaw 2017).

#### Actors of change

The primary actors of change in the region are farmers and tourists. We define farmer decision-making from attributes of ability and willingness to farm. Ability hereby refers to the conditioning factors of the farmer (e.g., age, agricultural training), while willingness addresses the farmer’s intentions and values, including cultural motives (Valbuena et al. 2010). These attributes are influenced by regional drivers of change which constrain and define periodic managerial decisions faced by farmers. Tourists are the second focal actor group, impacting the landscape by establishing a demand for specific (agri-)tourism services and playing a role in the valorization of the local landscape.

### Methodological overview

**Fig. 1:**
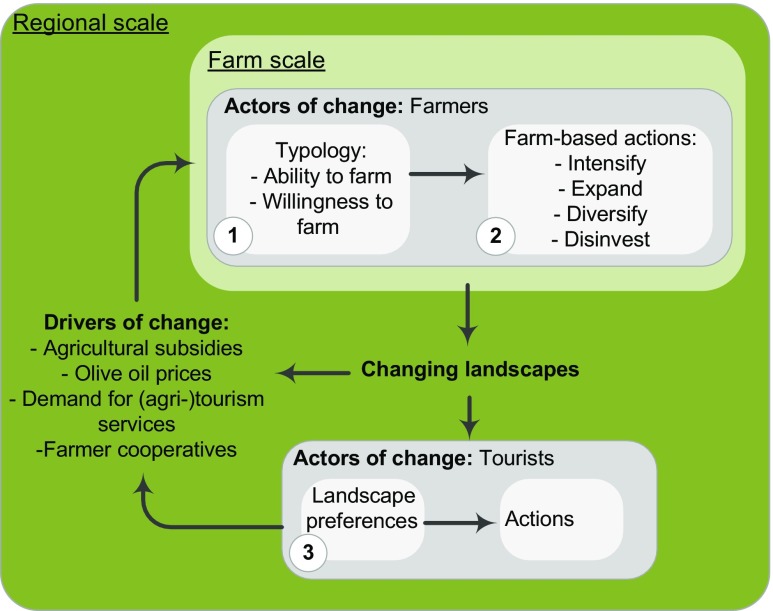
Research framework illustrating how our three objectives address characteristics and interactions between actors (farmers and tourists), regional drivers and landscape changes at multiple spatial scales (adapted from Valbuena et al. (2010))

Figure 1 illustrates how our three objectives address characteristics and interactions between actors (farmers and tourists), regional drivers and landscape changes at multiple spatial scales. The farmer typology developed in our first objective is based on structured interviews with 100 farmers analyzed through cluster analysis. The construction of actor typologies is a common means to study landscape dynamics (Bohnet 2008; Bohnet et al. 2011). Endogenous characteristics of actors (including motivational, managerial, or financial attributes) are identified and actors subsequently grouped to synthesize heterogeneous actions undertaken in response to drivers (Valbuena et al. 2010). Our second objective addresses how and if attributes of the identified farmer types explain past and expected individual actions on the landscape, contributing to regional landscape change. We build on the original framework by Valbuena et al. (2010) and add a second actor group (tourists), responding to landscape changes and influencing regional demand for (agri-)tourism services. Landscape preference studies have used simple rankings (Fyhri et al. 2009; Sayadi et al. 2005) as well as complex choice experiments (Hasund et al. 2011; Rambonilaza and Dachary-Bernard 2007). Our third objective, scoping landscape preferences of 63 tourists, is a first step in delineating how landscape users respond to and influence landscape change.

## Methods

### Farmer interviews

One-on-one interviews were carried out with 100 farmers in Gera between June and September 2015. Results obtained on farming system composition and management in our sample are largely in line with existing data, with over-representation of farmers managing arable land in addition to olive plantations (see Online Resource 1).

#### Surveying procedure

Respondents were approached in public spaces (bars, cafés, etc.) in the six villages of Gera: Mesagros, Palaiokipos, Papados, Perama, Plakados, and Skopelos. Further respondents were recruited via snowball sampling. The interviews lasted between 20 and 45 min. They used a seven-part structured questionnaire aiming to describe farmer behavior by investigating defining attributes of ability and willingness to farm (questionnaire sections outlined in Table 1, questionnaire in Online resource 2). Explanatory comments made by the respondents throughout the interview process were noted.

**Table 1:** Description and characterization of (1) variables used for clustering farmer types and (2) non-clustering variables analyzed statistically

	Survey question(s) used for variable derivation	Variable description and characterization	Data type	Determinant of ability/willingness/action
Clustering variables	D.1, D.2: “Professional engagement in agriculture”	(1) Full-time (2) Pluri-active (i.e., receiving additional incomes outside of sector, excluding pensions)	Binary	Farmer ability
	D.7: “Education”	(3) High school level diploma obtained (or equivalent)	Binary	Farmer ability
	D.8: “Agricultural training”	(4) Farmer has received formal agricultural training	Binary	Farmer ability
	G.1: “External knowledge usage”*	(5) Farmer makes use of cooperatives, the internet, consultants and/or research organizations when seeking advice on farm decision-making	Binary	Farmer ability
	D.9: “Social cooperative membership”	(6) Farmer is a member of a local, social cooperative	Binary	Farmer ability/farmer willingness
	H.4: “Perspective on future of local agricultural sector”*	(7) Farmer perspective is pessimistic (8) Farmer believes pluri-activity will be necessary	Binary	Farmer willingness
	D.5, H.3: “Cultural drive”*	(9) Farmer has inherited at least part of his land and has expressed both a desire to pass it onto successors and reluctance to sell	Binary	Farmer willingness
Non-clustering variables	D.3: “Farmer age”	-Farmer age	Continuous	Farmer ability
	D.10: “Subsidies received”	-Farmer receives subsidies (not including the Single Farm Payment (SFP))	Binary	Farmer ability
	D.9: “Traditional (non-social) cooperative membership”	-Farmer is a member of a local, traditional cooperative	Binary	Farmer ability
	G.1: “Internal knowledge usage”*	-Farmer makes use of own experience, experiences of neighbors and/or family members when seeking advice on farm decision-making	Binary	Farmer ability
	E: “Household composition”	-Farmer has successor working on the farm	Binary	Farmer ability/willingness
	H.2: “Influence of declining profits”	-Farmer refuses to ever quit farming despite consistently declining profits	Binary	Farmer willingness
	H.4: “Perspective on future of local agricultural sector”*	-Farmer perspective is optimistic	Binary	Farmer willingness
	C.1: “Past actions”*	-Farmer has intensified system in past -Farmer has expanded system in past -Farmer has diversified system in past -Farmer has disinvested system in past	Binary	Farm action
	H.1: “Future actions”*	-Farmer will intensify system in future -Farmer will expand system in future -Farmer will diversify system in future -Farmer will disinvest system in future	Binary	Farm action
	B.1: “Current farm area”	-Total farmland area	Continuous	Farm action
	B.4, B.5: “Current land use”*”	-Mix built -Mixed agriculture with no understory cultivation -Mixed agriculture with understory cultivation -Olive orchards and grazing land only -Olive orchards only	Categorical	Farm action
	B.6–B.10, E.1, F.1: “Current management intensity”*	-Farm management intensity	Continuous	Farm action
	B.5: “Organic production”	-Farm is certified organic	Binary	Farm action

Only the affirmative description is provided for binary (yes/no) variables

*Composite variables constructed from answers of multiple questions (survey question codes listed in Online Resource 2)

#### Analysis

Survey results from 100 valid interviews were initially explored using Pearson’s correlation. Variables describing the individual knowledge sources, actions on the farm, current land uses, and perspectives on the future of the local agricultural sector were each aggregated into overarching classes (see Table 1) to limit the number of variables for analysis (e.g., statements for past owned and/or rented area increase in survey section C.1 were both aggregated under “farmer has expanded system in past”). “Cultural drive” was also defined by multiple survey entries: willingness to pass land onto successors, unwillingness to loose ownership of land, and possession of inherited land. This characterization was substantiated by past research, in the region, stating the importance of land “not as assets, but as family capital and something you have to take care for the next generation” (Kizos et al. 2010), and elsewhere in Mediterranean traditional olive orchards (Duarte et al. 2008). “Management intensity” was characterized by use of fertilizers, pesticides, herbicides, irrigation, mechanization, tree density, marketing channel, terrace and stonewall maintenance, annual yield and agricultural income and hired and/or family labor, standardized to give a single summated score of intensity.

Nine variables (numbered in Table 1) were used as input for a hierarchical cluster analysis using complete linkage (furthest neighbor) with squared Euclidian distance, with the aim of grouping farmers in a typology based on individual attributes relating to ability and willingness to farm (following the criteria selection outlined in Valbuena et al. (2008)). The clustering variables addressed a farmer’s ability through their level of professional engagement in agriculture, education, experience from agricultural trainings and use of external knowledge sources. Farmer willingness was addressed through cultural drive and perspective on the future of the local agricultural sector. Social cooperative membership was used as a clustering variable determining both farmer ability and willingness, as these cooperatives serve as knowledge exchange platforms and promote landscape conservation and valorization. After cluster analysis, clustering variables and resulting farmer types were investigated via Discriminant Function Analysis (DFA), a commonly applied method for interpretation of clusters, indicating whether and how farmer types vary in relation to each clustering variable (Hair et al. 2010).

Additional non-clustering variables were tested for significant differences among farmer types. This served to both validate the derived clusters (Hair et al. 2010) (assessing how these newly revealed differences relate to the constructed types) and also evaluate if individual farmer attributes of ability and willingness in turn relate to differences in farm composition and farming strategies (actions outlined in Fig. 1). Depending on their data type, non-clustering variables were analyzed using the chi-square test, one-way ANOVA with Games-Howell post-hoc test or multiple regression analysis (see Table 1 for a comprehensive description and characterization of all clustering and non-clustering variables). For the investigation of regional driving forces, word counts were recorded identifying opinions on (traditional or social) cooperative membership and sentiments on current government support for the agricultural sector (within explanatory comments to survey questions D.2, D.9, D.10, F.1, H.4).

### Landscape preference survey

The landscape preference survey addresses landscape use complementarily to the analysis of farmer decision-making and landscape “production.” It investigates landscapes shaped by farmer actions, where disinvestments are associated with de-intensified landscapes and investments are portrayed in cultivated landscapes. Built landscapes were investigated to probe whether tourist preferences relate to elements of traditionality (seen in local architecture as well as in plantations) (Kianicka et al. 2006) and shed preliminary insight on the potential effect of built infrastructure as a result of increased off-farm activities. Coastal views, livestock, and other cultivations were excluded as these represent minor and non-rising farming strategies, or could provide bias. Landscapes were categorized following consultation of the academic literature, Panoramio and Google Maps Satellite and Street View imagery, exploratory field visits, and interviews with local scientific experts (complementing work from Beaufoy (2001); Fleskens (2008), see Online Resource 1). The approach aimed to distinguish landscapes at varying scales, so that preferences could be elicited towards view-sheds and immediately surrounding landscapes to define overall landscape perception (Karjalainen and Tyrväinen 2002).

Eighteen photos (shown in Online Resource 3) were selected following consultation with a local scientific expert. The photographs were subdivided into four sets. Sets 1 and 2 illustrate cultivated to progressively abandoned olive plantations, sets 3 and 4 show increasing housing sprawl and urbanization in forested areas. The duplication of sets for the same landscape change trajectory offered a validation mechanism for stated preferences. Each photograph within a set illustrates a different stage of the trajectory. To eliminate bias from terraces and slope, set 1 presents photos from non-sloping, non-terraced regions, while set 2 illustrates photos of sloping and terraced systems.

#### Surveying procedure

Interviews took place between June 22 and June 26 at the departure point of Mytilini Airport, engaging with 63 international and national tourists that had visited the island. On average, four international flights departed from the airport between 10 a.m. and 6 p.m. on each of the interviewing days in addition to national flights. The interviewer was present at least 2 h prior to the departures and approached all passengers in the waiting lounge. Although our sample does not allow for representativeness, the regularity of charter flights throughout the summer months make the selection of our sample adequate (see Online Resource 1). Interviews were carried out in English and lasted between 4 and 10 min. Respondents were initially asked to freely describe the landscapes of the island, and to specify which natural or cultural features they found particularly striking. Subsequently, they were presented with the first set of photographs in a randomized order and asked to rank them based on visual preference. Next, the respondents were asked to briefly state the motivation behind their election of most and least preferred choices. The exercise was repeated for the remaining sets. Questions on personal details followed and additional relevant comments made noted (questionnaire in Online Resource 2).

#### Analysis

Descriptive statistics were used to describe the tourist sample and the ranking score of each landscape photograph. The Wilcoxon Signed-Rank Test was used to verify consistency of means of matching photographs between coupled sets, validating the stated landscape preference or revealing potential sources of bias. A respondent-specific preferred ranking *order* score was calculated for each abandonment set, enabling an understanding of whether respondents ranked photographs in relation to the re-forestation/abandonment vs. cultivation construct (see Online Resource 1). These scores were investigated in terms of frequency distributions and Pearson’s correlation. Explanatory comments supporting ranking scores revealed additional insight on preferences. Open descriptions of the island were explored by means of word counts comparing mentions of natural (biophysical) vs. built features. Mentions of olive(tree)-related attributes were also counted. All statistical analyses was conducted in SPSS v23.

## Results

### Gera’s olive farmer typology: attributes of ability and willingness

The cluster analysis resulted in three farmer types characterized as disengaged farmers, active part-timers, and professional farmers (Table 2). DFA revealed Functions 1 and 2 (discriminating the groups) account for 89.1 and 18.1% of the variance with canonical correlations of 0.906 and 0.708, and eigenvalues 4.555 and 1.008, respectively. Function 1 discriminates disengaged farmers from the other two farmer types, while function 2 discriminates the active part-timers from the professional farmers. Professional engagement in farming (full-time vs. part-time or pluri-active) and education are the highest loading variables on discriminant function 2 and 1 respectively, representing important contributing variables to group separation. Test of equality of group means determined two clustering variables to be non-significantly different among groups (Table 2).

- *Disengaged* farmers constitute the predominant type within our sample. They are mostly part-time engaged in agriculture, with approximately one-third full-time farmers and only a few pluri-active farmers. This cluster represents farmers that have not obtained high-school level education. Their ability to farm is constrained by their part-time engagement in agriculture, lowest attendance to formal agricultural trainings and use of external knowledge sources. Their willingness to farm and maintain the cultivated landscape is also lower than that of the remaining identified groups; disengaged farmers rank lowest in social cooperative membership and cultural drive, and highest for pessimistic views on the future local agricultural sector.
- The *active part-timer* type is characterized by farmers with multiple income activities and a remaining group of part-timers mostly comprising retirees. Non-agricultural incomes derive from a wide range of sources: tourism, employment in the army, local shops and within fishery, forestry, construction, and education sectors. Active part-timer’s ability to farm is strengthened by high attendance to formal agricultural trainings and education. Few are social cooperative members, yet they are willing to keep farming for cultural motives, as this group has the highest proportion of culturally driven farmers. They believe pluri-activity will be successful and vital to the survival of the local agricultural sector.
- *Professional* farmers are mostly full-time farmers with no pluri-active members. They are the most educated, attend formal agricultural trainings and consult external sources, contributing to a high ability to farm. This group’s high willingness to farm and maintain the cultural landscape is demonstrated in the highest proportion of social cooperative members, strong cultural drive, and lowest share of pessimists.

**Table 2:** Distribution of farmers over (non-)clustering variables defining farmer ability and willingness to farm, listed per identified cluster group. Both DFA functions are significant at *p* < .001 (see Online Resource 1 for full results on non-clustering variables). Italicized entries indicate differences in variable values across cluster groups are non-significant. Underlined entries indicate variables most highly contributing to group separation

Cluster group	1	2	3			
Farmer type	Active part-timers	Disengaged farmers	Professional farmers	Equality of group means (significance)	DFA function 1	DFA function 2
No. of farmers	27	49	24			
% Composition per clustering variable						
(1) Full-time engagement in agriculture	0	35	75	.000	.021	−.673
(2) Pluri-active engagement in agriculture (i.e., receiving additional incomes outside of sector, excluding pensions)	67	16	0	.000	.107	.678
(3) High school diploma obtained (or equivalent)	82	6	100	.000	.756	−.160
(4) Farmer has received formal agricultural training	26	8	25	*.070*	.111	.022
(5) Farmer makes use of external knowledge sources	78	49	67	.039	.115	.098
(6) Farmer is a member of a local, social cooperative	11	2	29	.002	.146	−.201
(7) Farmer perspective is pessimistic on the future of the agricultural sector	56	61	42	*.294*	−.061	.093
(8) Farmer believes pluri-activity is necessary to maintain the future agricultural sector	93	76	63	.036	.010	.264
(9) Farmer is culturally driven	74	39	71	.002	.169	−.045
%* Composition per non-clustering variable				Sig.		
-Farmer age (*average age in years)	51	60	48	.003		
-Farmer receives subsidies (not including the SFP)	26	14	33	*.157*		
-Farmer is a member of a traditional (non-social) cooperative	37	41	33	*.863*		
-Farmer makes use of internal knowledge sources	93	98	92	*.508* ^a^		
-Farmer has successor working on the farm	15	12	17	*.931* ^a^		
-Farmer refuses to ever quit farming despite consistently declining profits	89	73	50	.007		
-Farmer perspective is optimistic	85	80	89	*.698* ^a^		

^a^Do not meet assumption for chi-square test, expected counts too low

Analysis of the farmer typology with non-clustering variables relating to ability and willingness partly validate the identified types and provide additional insight on farming dynamics (Table 2). Significant relationships were found between farmer typology and age (*F*(2, 97) = 6.162, *p* < .005) with older farmers in the disengaged group than in the active part-timer and professional groups. The average age of farmers interviewed was 54 years. The professional type holds the highest proportion of young farmers (30% <35, 68% <50), while disengaged farmers have an opposing distribution with 68% above 50. These relate to the clustering variables respectively attributing a high and low ability to farm to professional and disengaged farmers.

Traditional cooperative membership, presence of successors working on the farm, use of internal knowledge sources, and optimistic outlook did not vary significantly among farmer types. Over 80% of farmers in all types agreed with optimistic statements about the future of the local agricultural sector (lowest proportion amongst disengaged). Over 90% in all groups relied on internal knowledge sources (family members, neighbors, own experience) when undertaking farm-based decisions. Less than 20% of farmers in all groups had successors presently working on the farms. The majority of farmers in all groups decided not to subscribe to traditional cooperatives. Of these, over a quarter explicitly expressed criticism throughout the interview, as exemplified by Farmer 16 characterizing them “a total failure,” Farmer 41 as “deeply corrupted, cheat producers” and Farmer 17 stating they “do not function as cooperatives, rather as businesses.” Twenty-one percent of member farmers specified they were “passive” members, e.g., Farmer 31 stated cooperatives are only useful if viewed as being part of “tradition […] for storage, promotion, sales.”

A minority of farmers in all groups receive subsidies (excluding the SFP), particularly among disengaged farmers (14% alone are recipients), while enabling professionals’ ability to farm to greater extents (33% recipients). At least half of the farmers in all groups refuse to quit farming despite continuously declining profits, highest among active part-timers, confirming their strong willingness to farm due to cultural motives. Professionals are more reluctant to continue farming professionally under increasingly unprofitable conditions, despite also holding strong cultural motives. This is likely attributed to higher dependency on agricultural incomes. Culturally driven farmers expressed elements of pride by commenting on Gera’s high-quality olive oil (Farmer 26: “most prized”) and soil. They further recognized the role the olive sector plays in the maintenance of local cultural heritage (Farmer 38 referred to the preservation of monuments in olive fields) and the potential of linking these values to tourism (Farmer 1: “traditionality will make the difference”).

### Relating the farmer typology to past, present, and future actions on the farm

One-way ANOVA revealed a significant relation between farmer type on farming intensity (*F*(2, 97) = 4.337, *p* < .05) with higher intensity ratings among professionals than disengaged farmers. A significant regression model was found (*F*(9,86) = 3.37, *p* < .05, *R*^2^ = .26) when predicting the management intensity score from clustering variables, showing social cooperative membership (Beta = .25, *t*(86) = 2.35, *p* < .05), cultural drive (Beta = .21, *t*(86) = 2.09, *p* < .05) and full-time engagement in agriculture (Beta = .25, *t*(86) = 2.32, *p* < .05) as the significant predictors. Eight farmers explicitly acknowledged the important role of social cooperatives for “traditional” product promotion through novel certification schemes supporting integrated pest management and enabling access to new markets. Overall, 51% of farmers were classified as low-intensity, 40% as medium and 9% high intensity management. The professional group comprised more organic farmers than the others (*x*^2^(2) = 6.05, *p* < .05). Farm area and land use did not vary significantly among farmer types.

**Fig. 2:**
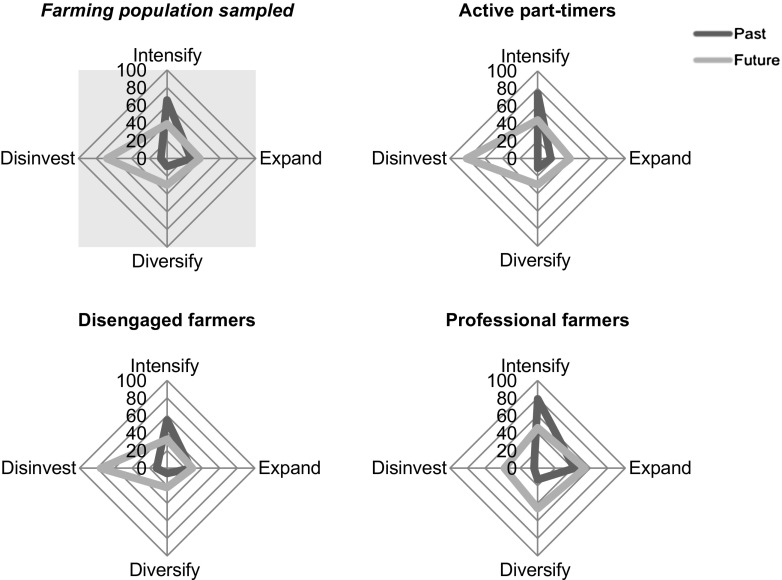
Frequency (%) of past decisions and expressed likelihood of future (dis)investments per farmer type and total farming population sampled. Past and future expressed likelihoods of decision are not exclusive

Figure 2 compares trends between past and intended actions amongst the farmer types. All types have similar patterns in past actions. Most frequent is intensification, followed by expansion and diversification with very few disinvestments. The disengaged type scores higher on past disinvestment than others, while professional farmers have the highest proportion of past investors. Disengaged farmers also have fewer cases of past intensification and diversification, while active-part timers the lowest proportion of past expansionists.

When comparing past and expressed likelihood of future actions, the most striking difference is the rise in the proportion of farmers expecting to disinvest. Disinvestment is anticipated within the next decade by 68% of interviewees (Fig. 2). Results revealed a higher frequency to disinvest amongst active part-timers (82% of total) than professionals (*x*^2^(2) = 13.79, *p* < .001). Eleven percent of farmers explicitly specified a lack of government support, declining (and poorly promoted) subsidies and the financial crisis have contributed to declining profits in the olive sector and thus farm investments. Farmer 48 states “sometimes the harvest costs are bigger than the product’s sale, so many people leave them [the olives] on the ground,” while Farmer 4 cannot afford consultancies and Farmer 61 stopped purchasing fertilizers. Further specifications relate to disinvestments occurring gradually, beginning on least accessible plots.

When investigating individual actions in isolation, the only short-term action to gain a positive mean score on the Likert scale (average 4.24, agreement scale 1 to 5) was “continue with current farming system.” Future and long-term *sectorial* sentiments however showed a prevailing agreement with positive statements of *change*, relating notably to olive oil markets becoming competitive again and conservation being recognized (positive outlook, section 3.1), with lowest mean Likert expressed for the continuation of abandonment (2.92). A discrepancy between present realities and expected future actions is witnessed also in the uptake of the farm by successors; only 14% of farmers have successors actively working on the farm, yet 76% agree with the statement: “When I retire, I will pass my land onto a successor.”

### Characteristics of interviewed tourists

Under the land zoning by Kizos and Koulouri (2006), results found 33% of respondents lodged in the eastern part of the island dominated by olives. Tourists combined beach-based activities with inland explorations. The most common activities were beach-based (over 75% of respondents), visiting towns and villages (57%) and hiking and trail walking (33%), in line with findings by Papanis and Kitrinou (2011). When asked to freely describe the environment(s) of Lesvos, 22% of respondents mentioned the presence of olive trees. While 81% of respondents referred to natural, or biophysical, attributes (e.g., topography, diversity in vegetation), only 11% referred to anthropogenic or built elements (e.g., villages, architectural styles), and 17% of respondents solely provided subjective descriptions (e.g., beautiful, interesting).

### Tourist landscape preferences: ranking scores and ranking order

The photographs with the highest mean ranking score per set, and thus preferred, were as following: (B) *intensively cultivated plantation* and (E) *traditionally cultivated plantation* for the abandonment sets, and (I) *mixed forest* and (P) *scattered housing* for housing sprawl sets. The photographs with the lowest mean score per set, and thus least preferred, were as follows: (D) and (H) *abandoned field*, (K) *scattered housing* and (R) *city* (Table 3). Preferred landscapes have lowest standard deviations in both sets. Results for urbanization (set 4) demonstrate a preference for natural or low-density housing landscapes, as the city landscape was least preferred by 75% of respondents. Results from set 3 differ, as the landscape depicting a densely built village received highest counts for both highest and lowest ranking scores. Validation of stated preferences for the abandonment sets (results of the Wilcoxon Signed-Rank Test) illustrated consistency in ranking position for abandoned and neglected plantations (43% of respondents marked abandonment in the same ranking position in both sets), but significant differences for traditional and intensive plantations. An analysis of ranking *order* revealed both sets 1 and 2 have a majority of respondents preferring cultivated landscapes and disfavoring abandoned ones (63.5% of interviewees in set 1 and 68.2% in set 2). Pearson’s correlation similarly revealed a positive relationship between preference score and frequency in set 2 (*r* = 0.639, *p* < .05) (see Online Resource 1).

**Table 3:** Standard deviation, mean, and median score per ranked landscape photograph, listed separately for sets illustrating processes of abandonment (sets 1 and 2) and housing sprawl (sets 3 and 4) in descending order of preference. Values for sets 1 and 2 are based on ranked scores with maximum values of 4 while sets 3 and 4 on maximum values of 5

	Photo ID	Photo description	Mean score	St. dev.	Median score
Ranked by mean average ranking—sets 1 and 2	B	Intensively cultivated plantation	3.06	0.93	3
	E	Traditionally cultivated plantation	3.00	1.12	3
	G	Neglected plantation	2.71	0.96	3
	C	Neglected plantation	2.67	0.97	2
	F	Intensively cultivated plantation	2.54	1.00	3
	A	Traditionally cultivated plantation	2.43	1.16	3
	D	Abandoned field	1.84	1.07	1
	H	Abandoned field	1.75	1.02	1
Ranked by mean average ranking—sets 3 and 4	P	Scattered housing	3.46	1.22	3
	N	Mixed forest	3.44	1.24	4
	O	Olive forest	3.40	1.26	3
	I	Mixed forest	3.24	1.29	3
	J	Olive forest	3.11	1.22	3
	Q	Suburb	3.11	1.23	3
	L	Sparsely built village	3.10	1.39	3
	M	Densely built village	3.06	1.77	3
	K	Scattered housing	2.49	1.27	3
	R	City	1.59	1.19	1

The ranking order results demonstrate a significant majority of tourists understand and value the landscape representations under the cultivation vs. abandonment construct. This is further exemplified in the justifications provided by the tourists for preferring cultivated landscapes. Tourists valued elements of typicality (Tourist 41 “I see Greece”), human influence and order. Abandoned landscapes were coherently appreciated by fewer tourists because they did not exhibit human influence. They were valued as more (and more diversely) vegetated, less monotonous, regulated and artificial. Responses of tourists that did not show consistency in their preference scores between first and second sets point towards some bias in the photographs (Tourists 3 and 16 stated preference based on “greenery” and 7 on “overall view preference”) while others stated preferences on impulse (Tourist 22 “very instinctive”). Correlating preference scores against covariates revealed no significant relationships.

Mean ranking scores and ranking order results for housing sprawl sets revealed preferences for an optimal level of housing. Only 25% of respondents preferred natural to built environments. Fewer (13%) respondents ranked both nature landscapes last and favored built environments. Explanatory descriptions for housing sprawl sets suggest preferences for a balance of both worlds, exemplified by Tourist 28 valuing the “contrast between nature and civilization, mountains and villages, rural and urban impressions.” While heavily built-up landscapes were seen as crowded and “too busy for a holiday” (Tourist 56), solely natural viewscapes were disfavored as they evoked negative sentiments of isolation and excluded traditional elements valued within local architecture.

## Discussion

### Linking actors, regional drivers, and landscape change trajectories

The identified farmer typology revealed a heterogeneous olive-farmer population with significantly different levels of ability and willingness to farm. Disengaged farmers (majority group) were defined by both low ability and willingness. Conversely, active part-timers and professional farmers have high ability and willingness, yet important differences in the two attributes exist between these farmer groups. Active-part timers revealed their high willingness to farm as strongly motivated by cultural (rather than solely profit-maximizing) reasons, as seen in their high cultural drive and refusal to stop farming despite steadily declining profits. Professional farmers, despite also stating a high cultural drive and thus refusing to loose ownership of their land, are less reluctant to quit farming as a profession when facing declining profits.

Typology results have implications for farm-level actions and (consequently) regional landscape change while revealing the influence of known regional drivers (section 1.1). Attributes and actions by each of the farmer types relate to a persistence of abandonment in the landscape, contrasting the stated landscape preferences of tourists as follows:

- Professional farmers’ dependency on agricultural incomes, coupled with a refusal to sell land is likely to either result in abandonment if profits decline past acceptable thresholds, or in an eventual transition towards pluri-activity driven by declining incomes rather than a desire to diversify (similar dynamics in Lamarque et al. (2013); Vernimmen et al. (2002)). A reliance of farming strategies upon sectorial profitability and cultural factors is also stated elsewhere in literature (Acosta et al. 2014; Sutherland 2010; Walther 1986), where a willingness to maintain land under family ownership and low presence of successors induces abandonment.
- Unlike professionals, active part-timers stated they will continue farming regardless of declining profits. Our results however demonstrate this group is also forecasting disinvestments. This action could be motivated by multiple factors. On the one hand, high average age of farmers in this group (as in the others) hinders ability and prospects of farm investments. On the other, lack of support and incentives towards this group by operating subsidies may also be acting as a deterrent.
- Disengaged farmers represented our samples’ predominant group despite low ability and willingness. Results indicate they do not wish to quit farming despite declining profits, yet they are pessimistic on the future of the sector, more willing to sell their land and more reluctant to pass it on to successors. Giourga et al. (2008) provide partial explanation to this phenomenon, suggesting poor infrastructural development and limited alternative employment opportunities are keeping (unwilling) farmers to the sector. Infrequent use of extension services by disengaged farmers presents a barrier for scaling up successful practices to increase profits. Their low management intensity and high frequency of disinvestments suggest a continuation of extensification and abandonment.

The willful maintenance of the cultivated landscape is thus supported by active-part timer and professional types, constrained by ability rather than willingness to farm. Their high willingness relates positively to tourists’ appreciation for olive plantations and nature-based activities, revealing prospects for reversing abandonment through actions of both actor groups. Our results show tourists value local rural settings because they provide elements of traditionality embedded within both cultivated-natural and built-up landscapes. This is in agreement with other tourist landscape preference studies, where results found highest appreciation within (agricultural) cultural landscapes (see Howley (2011); Hunziker et al. (2008); Schirpke et al. (2013)). In a study by Lamarque et al. (2013), farmers regarded themselves as stewards of the land and thus refused abandonment. Our results similarly saw farmers largely disagreeing with a statement forecasting continuation of abandonment, and revealed elements of land stewardship in the desire to pass land on to successors and actions of social cooperatives. These promote practices favoring conservation of the olive landscape, foster cultural farming motivations, and act as knowledge sharing platforms (Bock 2016; García-Martín et al. 2016; Shaw 2017).

Regional drivers of change reveal eventual mechanisms for interventions to maintain the cultivated landscape. Municipal, regional, and supranational policy may play a role in the development of suitable infrastructure, efficient subsidies and strategic marketing able to valorize local heritage in a culturally sustainable manner (Burton and Paragahawewa 2011; de Graaff et al. 2008; Mitchell and Barrett 2015). Subsidies could back farmer pluri-activity, supporting active part-timers in the maintenance of the agricultural-heritage landscape. A formal recognition of the heritage value in Gera’s farmed landscape (e.g., through High Nature Value Farmland designation) could further present novel opportunities for collaborations with existing cooperative or non-cooperative institutions, renewing an interest in the development of inland (agri-)tourism.

### Reflections on methods

Our approach did not aim to validate statements and discourses with additional data sources. Emphasis was intentionally placed on an analysis of characteristics, actions and preferences of two communities, and how these interact to influence regional landscape change. This decision reflects a need for sustainability sciences to move towards integrated assessments of socio-ecological systems, demanding depended explorations of how actors’ perceptions frame interactions with their environment, i.e., the “human feedback mechanisms” (Masterson et al. 2017). Further questions however remain. Our landscape preference investigation indicated some source of bias in the photographs and shed only preliminary insight from a limited sample. Future research may place additional emphasis on tourism behavior (eventually through a tourist typology), addressing the miss-match between landscape appreciation and use. While tourists favored cultivated landscapes, they often undertook activities in town centers and on the coast. Landscape preferences and activities of tourists contrast mass-tourism trends, yet past research has revealed the complexity inherent to the re-production/re-definition of space that comes with agri-tourism also (Figueiredo 2009; Galani-Moutafi 2013). Considerations on tourism development are especially relevant amidst a refugee crisis which may disincentivize investments.

## Conclusions

Our farmer typology and preliminary scoping of tourist landscape preferences are suited to inform discussions on the strategic planning of rural spaces, increasingly attentive to areas marked by agricultural abandonment (Soliva et al. 2008). The typology, based on attributes of ability and willingness to farm, confirms the importance of professional engagement in agriculture as a key determinant of group separation (Kizos et al. 2010). Importantly, however, the more comprehensive inclusion of multiple variables, specifically those relating to willingness to farm, explains differences in farm-based actions impacting the regional landscape. Our results revealed the prevalent farmer type is defined by low ability and willingness to farm, and is forecasting disinvestments resulting in further extensification of the landscape. A majority of farmers belonging to the active part-timer and professional types are however willing to maintain the cultivated landscape. An innovative aspect of our work was to relate investigations on different communities through methods often undertaken separately. We found a preference for cultivated over abandoned landscapes by a community often unaccounted for in landscape preference research (van Zanten et al. 2014). The tourism sector may provide options for further valorization of the public goods supplied by the cultivated landscape, supported by cultural farming motives and novel cooperative initiatives. Our assessment is valuable in its preliminary scoping for alternative futures explorations, where conflicting and synergetic dynamics between and amongst actors and landscape change are revealed.

## Electronic supplementary material

ESM 1 (DOCX 111 kb)

ESM 2 (DOCX 57.8 kb)

ESM 3 (DOCX 7.66 mb)
