# Supplementary material for: Perspectives of farmers and tourists on agricultural abandonment in east Lesvos, Greece
Source: Reg Environ Change. 2018 Feb 2;18(5):1467–79. doi: 10.1007/s10113-017-1276-4 (PMC6448353; doi:10.1007/s10113-017-1276-4)
Supplement: Supplementary file 1 — (DOCX 111 kb) [file 10113_2017_1276_MOESM1_ESM.docx]

**Online Resources 1 to “Perspectives of farmers and tourists on agricultural abandonment in east Lesvos, Greece”** *Regional Environmental Change*

Cecilia ZAGARIA^*^, [c.zagaria@vu.nl](mailto:c.zagaria@vu.nl); Catharina J. E. SCHULP; Thanasis KIZOS; Peter H. VERBURG

*Address of corresponding author: Environmental Geography Group, Institute for Environmental Studies, Vrije Universiteit Amsterdam, De Boelelaan 1087, 1081 HV Amsterdam, the Netherlands

Includes:

**Section A** – Comparison of surveyed farmer and tourist samples with existing (census) data

**Section B** – Review of classifications of olive plantation typologies relevant for construction of landscape preference survey

**Section C** – Calculation of ranking order scores from landscape preference survey

**Section D** – Comprehensive results of chi-square analysis with non-clustering variables from the farmer survey

**Section E** – Comprehensive results from Wilcoxon signed-rank test and analysis of preferred order scores from the landscape preferences survey

**Reference list to online resources**

1. **Comparison of surveyed farmer and tourist samples with existing (census) data**

*Surveyed farmers:*

**Table 1** Comparison of surveyed data from farmer interviews regarding farm system composition and management in Gera with existing data (ELSTAT, 2011; Giourga, Loumou, Tsevreni, & Vergou, 2008)

*Average farm size identified by Giourga et al. (2008) from a sample of 176 olive farmers in Lesvos was 8.2ha **Percentage of total Utilized Agricultural Area (UAA) is shown rather than percentage of farms with UAA

| **Farm system characteristic** | **Feature** | **Study sample**  **(% of farms interviewed)** | **Existing data**  **(% of farms with UAA)** |
| --- | --- | --- | --- |
| Farm land cover | Olive trees | 99 | 100 |
|  | Grazing | 21 | 11 |
|  | Arable | 36 | 5 |
|  | On-farm built infrastructure | 4 | N.D. |
| Size of farming system | Small-holders  (< 5ha) | 60 | N.D.* |
|  | Large farmland owners  (> 10ha) | 16 | N.D.* |
| Management inputs used | Fertilizer | 76 | 77 |
|  | Machinery | 72 | N.D. |
|  | Hired labor | 72 | N.D. |
|  | Pesticides | 24 | 30 |
|  | Herbicides | 26 | 30 |
|  | Irrigation | 38 | 3** |

*Surveyed tourists:*

63% of respondents interviewed were female and 37% were male. 27% of the respondents were below 29 years of age, 54% were in between 30 and 59 years and 19% above 60. Respondents resided in 15 different countries, the largest fractions in Germany (37%), Belgium (11%), the United Kingdom (11%) and Austria (10%). Despite the relatively low number of respondents, these sample characteristics match census data (ELSTAT, 2011) and that of other tourism studies on Lesvos (see Rontos et al. (2011)), where approximately 60% of respondents are female and international visitors are dominated by residents of the United Kingdom, Netherlands, Germany and Denmark (in addition to Turkish visitors arriving by ferry, these nationalities represent approximately 70% of visitors in 2012).

1. **Review of classifications of olive plantation typologies relevant for construction of landscape preference survey**

**Table 2** Defining visual attributes of existing olive plantation types at multiple scales, and establishing links with land use change trajectories

| **Land use change trajectory grouping** | **Plantation typology(ies)** | **Visual attributes** | | **Supporting literature** |
| --- | --- | --- | --- | --- |
|  |  | **(A) View-shed perspective** | **(B) Plot-level perspective** |  |
| Traditionality | (1) Low-input traditional  (2) Organic | -Steep/sloping land (hills, mountains)- marginal land  -Terraces  -Sparsely vegetated (low density, i.e. 20-50 trees/ha) | -Terraced  -No understory cultivation or cover due tillage/mowing  -Pruned  -Old/ancient (> 50 years)  -Large canopy | (Beaufoy, 2001; Fleskens, 2008; Kizos & Koulouri, 2010)  (Bieling & Bürgi, 2014; Kizos & Plieninger, n.d.) |
| Intensification | (1) Intensive traditional  (2) Organic | -Hilly/rolling plains  -Higher density than low-input traditional (80-250 trees/ha)  -Mix of younger and older trees  -May have understory cover/cultivation -May be terraced | -Younger (30 years)  -Pruned  -Understory may be cultivated  -May use irrigation  -May be terraced | (Beaufoy, 2001; Fleskens, 2008; Kizos & Koulouri, 2010) |
|  | (3) Intensive  modern | -Rolling/flat plains  -High density (200-4000 trees/ha)  -Allow mechanization; “vineyard alignment” | -Short-stem variety  -Younger trees (20 years)  -Drip irrigation  -No understory | (Beaufoy, 2001; Fleskens, 2008; Kizos & Koulouri, 2010) |
| Diversification | (1) Mixed cultivation  (2) Built (tourism) infrastructure | -Can include housing (may be traditional)  -Majority of olives removed and new tree species planted | -Can include housing  -Majority of olives removed and new tree species planted, cultivation may be present | (Kizos & Koulouri, 2010)  (Kizos & Plieninger, n.d.) |
| Extensification / abandonment | (1) Neglected  (2) Early abandonment  - 5 years | -Understory growth of bushes | -Understory growth of bushes  -Non-pruned trees – turning wild  -Neglected terrace | (Kizos, Dalaka, & Petanidou, 2010; Kizos & Koulouri, 2010; Kizos & Plieninger, n.d.) |
|  | (3) Abandoned  - 25 years | -Dense forest, including oak, pines, maquis | -Trees have “turned wild”, branches sprouting all over trunk, less foliage, “bushy” appearance | (Kizos et al., 2010; Kizos & Koulouri, 2010; Kizos & Plieninger, n.d.) |

1. **Calculation of ranking order scores from landscape preference survey**

The preferred photograph was assigned a highest possible score of 4 (5 for sets 3 and 4) and so on in descending order of preference. Order scores account for the ranking across a whole set of photos, and ranged from a lowest possible value per sequence of 20 (sequence A,B,C,D / E,F,G,H) and a highest of 30 for the reversed order (sequence D,C,B,A / H,G,F,E) (see **Figure 1**). Scores in the range of 20 to 22 indicate a strong preference for cultivated over wild landscapes (as they comprised sequences which ranked both cultivated landscapes over the two re-wilding, abandoned landscapes) while scores ranging 28 to 30 indicated the opposite preference for wild landscapes over cultivated.

**Fig. 1** Example calculation of respondents' ranking order preference score for set 1

1. **Comprehensive results of chi-square analysis with non-clustering variables from the farmer survey**

Significant associations were found between farmer type and:

- Farmers refusing to ever quit farming despite consistently declining profits (*X*^2^(2) = 9.64, *p* < .05)

No significant associations were found between farmer type and:

- Farmer receiving subsidies (not including the SFP) (*X*^2^(2) = 3.74, *p* = .157)
- Farmer being a member of a traditional (non-social) cooperative (*X*^2^(2) = 0.40, *p* = .863)

Three variables did not meet the require assumption for the chi-square test, expected counts too low:

- Farmer makes use of internal knowledge sources (*X*^2^(2) = 1.79, *p* = .508)
- Farmer has a successor working on the farm (*X*^2^(2) = 0.28, *p* = .931)
- Farmer perspective on the future of the local sector is optimistic (*X*^2^(2) = 0.84, *p* = .698)

1. **Comprehensive results from Wilcoxon signed-rank test and analysis of preferred order scores from the landscape preferences survey**

*Results from Wilcoxon signed-rank test (landscape preference survey):*

Traditional plantations were ranked with significantly higher scores in the first set as opposed to the second (*z* = -3.183, *r* = -0.40), while intensive systems were scored higher in the second set (*z* = -2.816, *r* = -0.35) (**Table 3**). For the sets illustrating housing sprawl (sets 3 and 4, **Table 4**), the two forested systems with no built infrastructure were similarly ranked (*r* = -0.17 and -0.22 respectively for mixed and olive forests). Statistically significant differences were found for scattered housing and densely built village / city (*z* = -3.987 and -5.015, *r* = -0.50 and -0.63 respectively.

**Table 3** Analysis of means for set 1 and set 2

| **Photo ID and description** | **Count negative**  Set 1 > Set 2 | **Count positive**  Set 1 < Set 2 | **Count neutral**  Set 1 = Set 2 | *z* | *p* | *r* |
| --- | --- | --- | --- | --- | --- | --- |
| *(A/E) Traditional* | 35 | 11 | 17 | -3.183 | 0.001 | -0.40 |
| *(B/F) Intensive* | 18 | 32 | 13 | -2.816 | 0.005 | -0.35 |
| *(C/G) Neglected* | 22 | 13 | 22 | -0.261 | 0.794 | -0.03 |
| *(D/H) Abandoned* | 19 | 17 | 27 | -0.780 | 0.436 | -0.10 |

**p* (Asymp. Sig. (2-tailed)) < 0.05 signifies the same system was ranked significantly differently in the two sets

** *r* >/= to 0.5 signifies a large change between the two rankings of the same system; an *r*-value closer to 0.3 signifies a medium change and a value < 0.3 a small change.

**Table 4** Analysis of means for set 3 and set 4

| **Photo ID and description** | **Count negative**  Set 3 > Set 4 | **Count positive**  Set 3 < Set 4 | **Count neutral**  Set 3 = Set 4 | *z* | *p* | *r* |
| --- | --- | --- | --- | --- | --- | --- |
| *(I/N) Mixed forest* | 26 | 17 | 20 | -1.363 | 0.173 | -0.17 |
| *(J/O) Olive forest* | 28 | 18 | 17 | -1.740 | 0.082 | -0.22 |
| *(K/P) Scattered housing* | 38 | 14 | 1 | -3.987 | 0.000 | -0.50 |
| *(L/Q) Sparsely built villages/suburb* | 25 | 19 | 19 | -0.042 | 0.967 | -0.01 |
| *(M/R) Densely built villages/ city* | 5 | 37 | 21 | -5.015 | 0.000 | -0.63 |

**p* (Asymp. Sig. (2-tailed)) < 0.05 signifies the same system was ranked significantly differently in the two sets

** *r* >/= to 0.5 signifies a large change between the two rankings of the same system; an *r*-value closer to 0.3 signifies a medium change and a value < 0.3 a small change.

*Frequency of preference order scores (landscape preference survey):*

**Figure 2** Frequency (%) of classified ranked sequence scores for abandonment sets 1 and 2; scores were derived for each respondent based on the order in which they ranked landscapes, assessing whether this followed the abandonment gradient illustrated (see Online Resource C for details on calculation of ranked sequence/order preference scores)

**Reference list to Online Resources 1**

Beaufoy, G. (2001). The Environmental Impact of Olive oil Production in the European Union: Practical Options for Improving the Environmental Impact, *5210*, 0–73. Retrieved from http://ec.europa.eu/environment/agriculture/pdf/oliveoil.pdf

Bieling, C., & Bürgi, M. (2014). *D3.1 List and documentation of case study landscapes selected for HERCULES*. Retrieved from http://www.hercules-landscapes.eu/tartalom/HERCULES_WP3_D3_1_ALUFR_final.pdf

ELSTAT. (2011). Hellenic Statistical Authority. Retrieved from www.statistics.gr

Fleskens, L. (2008). A typology of sloping and mountainous olive plantation systems to address natural resources management. *Annals of Applied Biology*, *153*(3), 283–297. https://doi.org/10.1111/j.1744-7348.2008.00260.x

Giourga, C., Loumou, A., Tsevreni, I., & Vergou, A. (2008). Assessing the sustainability factors of traditional olive groves on Lesvos Island, Greece (Sustainability and traditional cultivation). *GeoJournal*, *73*(2), 149–159. https://doi.org/10.1007/s10708-008-9195-z

Kizos, T., Dalaka, A., & Petanidou, T. (2010). Farmers’ attitudes and landscape change: evidence from the abandonment of terraced cultivations on Lesvos, Greece. *Agriculture and Human Values*, *27*(2), 199–212. https://doi.org/10.1007/s10460-009-9206-9

Kizos, T., & Koulouri, M. (2010). Same Land Cover, Same Land Use at the Large Scale, Different Landscapes at the Small Scale: Landscape Change in Olive Plantations on Lesvos Island, Greece. *Landscape Research*, *35*(4), 449–467. https://doi.org/10.1080/01426390802048297

Kizos, T., & Plieninger, T. (n.d.). Agroforestry systems change in the Mediterranean: Some evidence from Greek and Spanish examples. JOUR. Retrieved from http://www.geo.aegean.gr/earth-conference2008/papers/papers/A08ID185.pdf

Rontos, K., Papanis, E., & Kitrinou, E. (2011). Analysing tourist’s profile, holidays’ characteristics and perceived tourist cost at Lesvos Island, Greece. *Journal of Management Sciences and Regional Development*, (7). Retrieved from http://www.stt.aegean.gr/geopolab/GEOPOL PROFILE.htm
