# Supplementary material for: Perspectives of farmers and tourists on agricultural abandonment in east Lesvos, Greece
Source: Reg Environ Change. 2018 Feb 2;18(5):1467–79. doi: 10.1007/s10113-017-1276-4 (PMC6448353; doi:10.1007/s10113-017-1276-4)
Supplement: Supplementary file 2 — (DOCX 57.8 kb) [file 10113_2017_1276_MOESM2_ESM.docx]

**Online Resource 2 to “Perspectives of farmers and tourists on agricultural abandonment in east Lesvos, Greece”** *Regional Environmental Change*

Cecilia ZAGARIA^*^, [c.zagaria@vu.nl](mailto:c.zagaria@vu.nl); Catharina J. E. SCHULP; Thanasis KIZOS; Peter H. VERBURG

*Address of corresponding author: Environmental Geography Group, Institute for Environmental Studies, Vrije Universiteit Amsterdam, De Boelelaan 1087, 1081 HV Amsterdam, the Netherlands

Includes:

**Section A** – Survey investigating farmer decision-making

**Section B** – Landscape preference survey with tourists

1. **Survey investigating farmer decision-making**

***Part A- Farm and household location***

| **A.1** | Please locate your farming plot(s) on the map provided  (by delineating polygons if possible) |
| --- | --- |

| **A.2** | Please locate your residence on the map provided, and mark it with the letter “H” |
| --- | --- |

***Part B- Farm characteristics***

| **B.1** | What is the total size of your farmland? | …………………… (ha) |
| --- | --- | --- |

| **B.2** | What is the ownership status of your farm? | (1) Owned | (2) Rented | (3) Both |
| --- | --- | --- | --- | --- |

| **B.3** | What is the length of your initial farm activity? | …………………… (years) |
| --- | --- | --- |

| **B.4** | Can you give an approximate % of the total area cover share of each system within your farm? | (1) Olive groves  …..% | (2) Arable  …..% | (3) Grazing  …..% | (4) Agro-tourism  …..% | (5) Other- please specify:  …..% |
| --- | --- | --- | --- | --- | --- | --- |

| **B.5** | Which of the following are present or apply to your farming system? | (1) Sheep | (2) Goats | (3) Terraces | |
| --- | --- | --- | --- | --- | --- |
|  |  | (4) Understory cultivation | | (5) Organic farming | (6) Other- please specify: |

| **B.6** | What farm management is being practiced? | (1) Pruning | (2) Harvesting | (3) Terrace maintenance |
| --- | --- | --- | --- | --- |
|  |  | (4) Understory clearance | | (5) Other- please specify: |

| **B.7** | What farm inputs are being used? | (1) Hired labor | (2) Pesticides | (3) Herbicides | | (4) Fertilizers |
| --- | --- | --- | --- | --- | --- | --- |
|  |  | (5) Irrigation | (6) Machinery | | (7) Other- please specify: | |

| **B.8** | What is your tree and grazing density? | (1) Tree  ………. (trees/ha) | (2) Grazing  ………. (animals/ha) |
| --- | --- | --- | --- |

| **B.9** | What is your produce used for? | (1) Own consumption | (2) Sale to local market | (3) Sale for national export | (4) Sale for international export | (5) Other- please specify: |
| --- | --- | --- | --- | --- | --- | --- |

| **B.10** | What is your yield per produce? | (1) Olive oil  ………..  (l/ha/year) | (2) Table olives  ………..  (kg/ha/year) | (3) Other produce- please specify:  ……….. | (4) Other produce- please specify:  ……….. |
| --- | --- | --- | --- | --- | --- |

| **B.11** | How does your farming system vary across your different plots? | | |
| --- | --- | --- | --- |
| **B.11.1** | Land use | (1) Differs | (2) Does not differ |
| **B.11.2** | Density | (1) Differs | (2) Does not differ |
| **B.11.3** | Maintenance | (1) Differs | (2) Does not differ |
| **B.11.4** | Inputs and management practices | (1) Differs | (2) Does not differ |
| **B.11.5** | Other- please specify: | | |

| **B.12** | (A) To what extent do the following factors influence the differences across your plots? | 1 | 2 | 3 | 4 | 5 | (B) Please explain how: |
| --- | --- | --- | --- | --- | --- | --- | --- |
|  |  | Strongly disagree | Disagree | Neutral | Agree | Completely agree |  |
| **B.12.1** | Elevation | ⃝ | ⃝ | ⃝ | ⃝ | ⃝ |  |
| **B.12.2** | Soil suitability | ⃝ | ⃝ | ⃝ | ⃝ | ⃝ |  |
| **B.12.3** | Land value | ⃝ | ⃝ | ⃝ | ⃝ | ⃝ |  |
| **B.12.4** | Ownership status | ⃝ | ⃝ | ⃝ | ⃝ | ⃝ |  |
| **B.12.5** | Proximity to home | ⃝ | ⃝ | ⃝ | ⃝ | ⃝ |  |
| **B.12.6** | Availability of capital | ⃝ | ⃝ | ⃝ | ⃝ | ⃝ |  |
| **B.12.7** | Proximity to urban center | ⃝ | ⃝ | ⃝ | ⃝ | ⃝ |  |
| **B.12.8** | Profitability | ⃝ | ⃝ | ⃝ | ⃝ | ⃝ |  |
| **B.12.9** | Accessibility | ⃝ | ⃝ | ⃝ | ⃝ | ⃝ |  |
| **B.12.10** | Farm system of previous owner/tenant | ⃝ | ⃝ | ⃝ | ⃝ | ⃝ |  |
| **B.12.11** | Plot area | ⃝ | ⃝ | ⃝ | ⃝ | ⃝ |  |
| **B.12.12** | Age of tree crops | ⃝ | ⃝ | ⃝ | ⃝ | ⃝ |  |
| **B.12.13** | Other-please specify: | ⃝ | ⃝ | ⃝ | ⃝ | ⃝ |  |

***Part C- Past changes to your farming system***

| **C.1** | (A) How has your system changed since the beginning of your ownership/tenancy? | | | (B) In what way?  (e.g. increased, decreased, invested etc.) |
| --- | --- | --- | --- | --- |
| **C.1.1** | Land use | (1) Changed | (2) Did not change |  |
| **C.1.2** | Density | (1) Changed | (2) Did not change | (trees/animals / ha) |
| **C.1.3** | Maintenance | (1) Changed | (2) Did not change |  |
| **C.1.4** | Irrigation, mechanization, input management | (1) Changed | (2) Did not change |  |
| **C.1.5** | Other management practices | (1) Changed | (2) Did not change |  |
| **C.1.6** | Owned land area | (1) Changed | (2) Did not change | (ha) |
| **C.1.7** | Rented land area | (1) Changed | (2) Did not change | (ha) |
| **C.1.8** | Other- please specify: | | | |

***Part D- Personal details***

| **D.1** | Are you a full-time farmer? | (1) Full-time | (2) Part-time |
| --- | --- | --- | --- |

| **D.2** | Do you have any additional income apart from your agricultural production? | (1) Subsidies | (2) Tourism | (3) No other income source | (4) Other-please specify: |
| --- | --- | --- | --- | --- | --- |

| **D.3** | What is your age? | ……. years |
| --- | --- | --- |

| **D.4** | What is your gender? | (1) Male | (2) Female |
| --- | --- | --- | --- |

| **D.5** | Was your farm inherited or purchased? | (1) Inherited | (2) Purchased | (3) Both |
| --- | --- | --- | --- | --- |

| **D.6** | Do you reside in Gera all year round? | (1) All-year | (2) Other- please specify: |
| --- | --- | --- | --- |

| **D.7** | What is your highest education level? | (1) Primary school | (2) Middle school | (3) High school |
| --- | --- | --- | --- | --- |
|  |  | (4) University | | (5) Other- please specify: |

| **D.8** | Where have you received your agricultural training? | …………………………………………………………… |
| --- | --- | --- |

| **D.9** | Are you a member of a farming cooperative? | 1. Yes | 1. No | (3) State name if applicable:  ……………. |
| --- | --- | --- | --- | --- |

| **D.10** | Which, if any, subsidy do you receive? | (1) Single Farm Payment | (2) Less Favored Area | (3) Regulation of Aegean Islands | (4) Rural Development- Please specify: |
| --- | --- | --- | --- | --- | --- |

***Part E- Household composition***

|  | What is your household composition? | | | | | | | |
| --- | --- | --- | --- | --- | --- | --- | --- | --- |
|  | Family member | (A) Gender | (B) Age | (C) Work in the farm | (D Public servant | (E) Private sector | (F) Pension | (G) Student |
| **E.1.1** | Spouse /husband |  |  |  |  |  |  |  |
| **E.1.2** | Child |  |  |  |  |  |  |  |
| **E.1.3** | Child |  |  |  |  |  |  |  |
| **E.1.4** | Child |  |  |  |  |  |  |  |
| **E.1.5** | Parent |  |  |  |  |  |  |  |
| **E.1.6** | Parent |  |  |  |  |  |  |  |
| **E.1.7** | Other |  |  |  |  |  |  |  |
| **E.1.8** | Other |  |  |  |  |  |  |  |

***Part F- Household income***

|  | What is your monthly household income per sector? (€) | | | | | | |  |
| --- | --- | --- | --- | --- | --- | --- | --- | --- |
|  | Income source | (A)  <100 | (B)  100-500 | (C)  500-1000 | (D)  1000-2000 | (E)  2000-4000 | (F)  4000-6000 | (G)  >6000 |
| **F.1.1** | Agriculture (excluding subsidies) |  |  |  |  |  |  |  |
| **F.1.2** | Subsidies |  |  |  |  |  |  |  |
| **F.1.3** | Tourism |  |  |  |  |  |  |  |
| **F.1.4** | Wages (public) |  |  |  |  |  |  |  |
| **F.1.5** | Wages (private) |  |  |  |  |  |  |  |
| **F.1.6** | Pensions |  |  |  |  |  |  |  |
| **F.1.7** | Other- please specify: |  |  |  |  |  |  |  |

***Part G- Advice on decision-making***

| *When making key decisions for my farm, I consult …* | | 1 | 2 | 3 | 4 | 5 |
| --- | --- | --- | --- | --- | --- | --- |
|  |  | Strongly disagree | Disagree | Neutral | Agree | Completely agree |
| **G.1.1** | My farming cooperative/association | ⃝ | ⃝ | ⃝ | ⃝ | ⃝ |
| **G.1.2** | The internet | ⃝ | ⃝ | ⃝ | ⃝ | ⃝ |
| **G.1.3** | My family | ⃝ | ⃝ | ⃝ | ⃝ | ⃝ |
| **G.1.4** | Private consultants | ⃝ | ⃝ | ⃝ | ⃝ | ⃝ |
| **G.1.5** | Research organizations | ⃝ | ⃝ | ⃝ | ⃝ | ⃝ |
| **G.1.6** | I base decisions on experiences of neighboring farms | ⃝ | ⃝ | ⃝ | ⃝ | ⃝ |
| **G.1.7** | I base decisions on my own expertise | ⃝ | ⃝ | ⃝ | ⃝ | ⃝ |
| **G.1.8** | Other- please specify: | ⃝ | ⃝ | ⃝ | ⃝ | ⃝ |

***Part H- Future of your farm and local agricultural sector***

| *In ten years’ time, I expect to …* | | 1 | 2 | 3 | 4 | 5 |
| --- | --- | --- | --- | --- | --- | --- |
|  |  | Strongly disagree | Disagree | Neutral | Agree | Completely agree |
| **H.1.1** | Be continuing with my current farming system | ⃝ | ⃝ | ⃝ | ⃝ | ⃝ |
| **H.1.2** | Have expanded my farm area | ⃝ | ⃝ | ⃝ | ⃝ | ⃝ |
| **H.1.3** | Have diversified my farming system | ⃝ | ⃝ | ⃝ | ⃝ | ⃝ |
| **H.1.4** | Have invested in new technologies | ⃝ | ⃝ | ⃝ | ⃝ | ⃝ |
| **H.1.5** | Have sold/stop leasing some of my current land | ⃝ | ⃝ | ⃝ | ⃝ | ⃝ |
| **H.1.6** | Have reached my retirement age and stopped farming | ⃝ | ⃝ | ⃝ | ⃝ | ⃝ |
| **H.1.7** | Have found alternative employment | ⃝ | ⃝ | ⃝ | ⃝ | ⃝ |
| **H.1.8** | Have invested in my own agro-tourism business | ⃝ | ⃝ | ⃝ | ⃝ | ⃝ |
| **H.1.9** | Have taken up early retirement | ⃝ | ⃝ | ⃝ | ⃝ | ⃝ |
| **H.1.10** | No longer be farming | ⃝ | ⃝ | ⃝ | ⃝ | ⃝ |
| **H.1.11** | Become a part-time farmer | ⃝ | ⃝ | ⃝ | ⃝ | ⃝ |
| **H.1.12** | Have abandoned my land | ⃝ | ⃝ | ⃝ | ⃝ | ⃝ |
| **H.1.13** | Other- please specify: | ⃝ | ⃝ | ⃝ | ⃝ | ⃝ |

| **H.2** | After how many years of declining agricultural profits would you stop farming (and seek alternative employment)? | (1) 2 years | (2) 4 years | (3) 6 years | (4) Other-please specify: |
| --- | --- | --- | --- | --- | --- |

| *When I retire, I will …* | | 1 | 2 | 3 | 4 | 5 |
| --- | --- | --- | --- | --- | --- | --- |
|  |  | Strongly disagree | Disagree | Neutral | Agree | Completely agree |
| **H.3.1** | Be 65 years of age | ⃝ | ⃝ | ⃝ | ⃝ | ⃝ |
| **H.3.2** | Pass my farm onto a successor | ⃝ | ⃝ | ⃝ | ⃝ | ⃝ |
| **H.3.3** | Sell my land | ⃝ | ⃝ | ⃝ | ⃝ | ⃝ |
| **H.3.4** | Stop the lease of my land | ⃝ | ⃝ | ⃝ | ⃝ | ⃝ |
| **H.3.5** | Abandon my land | ⃝ | ⃝ | ⃝ | ⃝ | ⃝ |
| **H.3.6** | Rent out my land | ⃝ | ⃝ | ⃝ | ⃝ | ⃝ |
| **H.3.7** | Other- please specify: | ⃝ | ⃝ | ⃝ | ⃝ | ⃝ |

| How do the following statements agree with your vision of the future local agricultural sector? | | 1 | 2 | 3 | 4 | 5 |
| --- | --- | --- | --- | --- | --- | --- |
|  |  | Strongly disagree | Disagree | Neutral | Agree | Completely agree |
| **H.4.1** | *I believe that olive oil production can be competitive in global markets again* | ⃝ | ⃝ | ⃝ | ⃝ | ⃝ |
| **H.4.2** | *I believe that young people will seek employment outside of the agricultural sector* | ⃝ | ⃝ | ⃝ | ⃝ | ⃝ |
| **H.4.3** | *I believe that abandonment of olive oil production will continue* | ⃝ | ⃝ | ⃝ | ⃝ | ⃝ |
| **H.4.4** | *I believe that pluri-activity (having more than one job) can be combined with olive oil production* | ⃝ | ⃝ | ⃝ | ⃝ | ⃝ |
| **H.4.5** | *I believe that olive cultivation will be active in the area only if farmers have extra incomes (e.g. from tourism)* | ⃝ | ⃝ | ⃝ | ⃝ | ⃝ |
| **H.4.6** | *I believe that there were opportunities for young people to be involved in farming, but many did not utilize them* | ⃝ | ⃝ | ⃝ | ⃝ | ⃝ |
| **H.4.7** | *I believe that the local importance of olive plantations will be recognized for their protection* | ⃝ | ⃝ | ⃝ | ⃝ | ⃝ |

1. **Landscape preference survey with tourists**

General questions:

| **A.1** | How would you describe the landscape of the island? Any specific cultural or natural features which you found striking? |  |
| --- | --- | --- |

Ranking exercise:

|  | **Ranking** | Photo | | | |
| --- | --- | --- | --- | --- | --- |
|  |  | A | B | C | D |
| **B.1** | 1- Least preferred |  |  |  |  |
| **B.2** | 2 |  |  |  |  |
| **B.3** | 3 |  |  |  |  |
| **B.4** | 4- Most preferred |  |  |  |  |

*Please rank the photos provided in order of your personal preference*

|  | **Ranking** | Photo | | | |
| --- | --- | --- | --- | --- | --- |
|  |  | E | F | G | H |
| **C.1** | 1- Least preferred |  |  |  |  |
| **C.2** | 2 |  |  |  |  |
| **C.3** | 3 |  |  |  |  |
| **C.4** | 4- Most preferred |  |  |  |  |

|  | **Ranking** | Photo | | | | |
| --- | --- | --- | --- | --- | --- | --- |
|  |  | N | O | P | Q | R |
| **E.1** | 1- LP |  |  |  |  |  |
| **E.2** | 2 |  |  |  |  |  |
| **E.3** | 3 |  |  |  |  |  |
| **E.4** | 4 |  |  |  |  |  |
| **E.5** | 5-MP |  |  |  |  |  |

|  | **Ranking** | Photo | | | | |
| --- | --- | --- | --- | --- | --- | --- |
|  |  | I | J | K | L | M |
| **D.1** | 1- LP |  |  |  |  |  |
| **D.2** | 2 |  |  |  |  |  |
| **D.3** | 3 |  |  |  |  |  |
| **D.4** | 4 |  |  |  |  |  |
| **D.5** | 5-MP |  |  |  |  |  |

Explaining preferences:

|  | Briefly explain why you chose each of your highest and lowest ranked photographs | | |
| --- | --- | --- | --- |
|  |  | 1. HIGHEST | 1. LOWEST |
| **F.1** | Group B |  |  |
| **F.2** | Group C |  |  |
| **F.3** | Group D |  |  |
| **F.4** | Group E |  |  |

Ease of choice:

|  | How would you describe the ease with which you made these choices? | 1 | 2 | 3 | 4 | 5 |
| --- | --- | --- | --- | --- | --- | --- |
| **G.1** |  | Very difficult | Relatively difficult | Neutral | Relatively easy | Very easy |
|  |  |  |  |  |  |  |

Personal details:

| **H.1** | Age | (1)  < 18 | (2)  19-29 | (3)  30-39 | (4)  40-49 |
| --- | --- | --- | --- | --- | --- |
|  |  | (5)  50-59 | (6)  60-69 | (7)  >70 | |

| **H.2** | Gender | (1) Male | (2) Female |
| --- | --- | --- | --- |

| **H.3** | Country of residence | ……………………………………………… |
| --- | --- | --- |

| **H.4** | Type of permanent residence location | (1)  Urban | (2)  Suburban | (3)  Rural |
| --- | --- | --- | --- | --- |

| **H.5** | Occupation | ……………………………………………… |
| --- | --- | --- |

| **H.6** | What tourism activities did you mostly participate in? Please tick **top-3** in terms of time spent | (1)  Beach and sea-based  (including fishing, sailing) | (2)  Visiting local towns and villages | (3)  Walking/hiking through countryside or inland nature trails | (4)  Bird-watching |
| --- | --- | --- | --- | --- | --- |
|  |  | (5)  Agro-tourism-based | (6)  Visits to archaeological sites | (7)  Other, please specify:  ……………………………………… | |

| **H.7** | In which town/city did you accommodate? | ……………………………………………… |
| --- | --- | --- |

| **H.8** | What was the length of your stay? | ……………………………………………… |
| --- | --- | --- |

| **H.9** | Was this your first time visiting the island? | 1. Yes | 1. No |
| --- | --- | --- | --- |
