# Supplementary material for: Perspectives of farmers and tourists on agricultural abandonment in east Lesvos, Greece
Source: Reg Environ Change. 2018 Feb 2;18(5):1467–79. doi: 10.1007/s10113-017-1276-4 (PMC6448353; doi:10.1007/s10113-017-1276-4)
Supplement: Supplementary file 3 — (DOCX 7.66 mb) [file 10113_2017_1276_MOESM3_ESM.docx]

**Online Resources 3 to “Perspectives of farmers and tourists on agricultural abandonment in east Lesvos, Greece”** *Regional Environmental Change*

Cecilia ZAGARIA^*^, [c.zagaria@vu.nl](mailto:c.zagaria@vu.nl); Catharina J. E. SCHULP; Thanasis KIZOS; Peter H. VERBURG

*Address of corresponding author: Environmental Geography Group, Institute for Environmental Studies, Vrije Universiteit Amsterdam, De Boelelaan 1087, 1081 HV Amsterdam, the Netherlands

Includes:

**Section A** – Landscape photos ranked by tourists during the preference survey

1. Landscape photos ranked by tourists during the preference survey

**Agricultural abandonment: Set 1 Set 2**


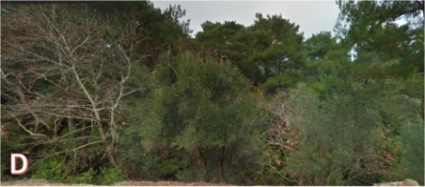

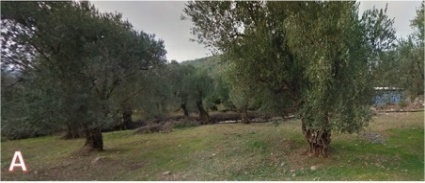

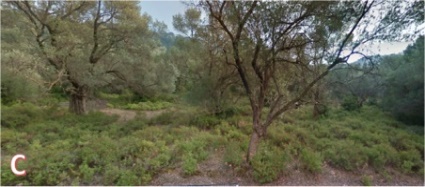

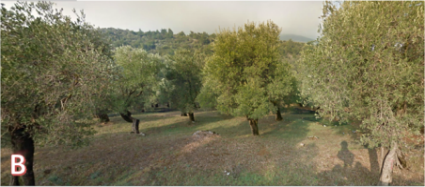

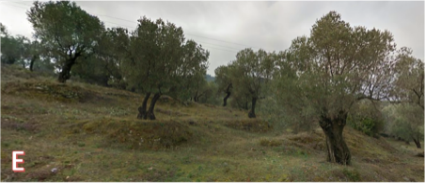

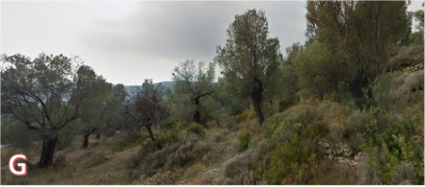

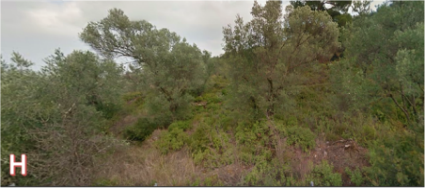

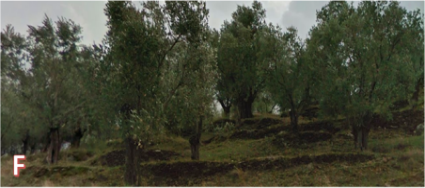


Low-input traditional

Intensive

Neglected

Abandoned

© 2015 Google

**Housing sprawl / urbanization: Set 3 Set 4**


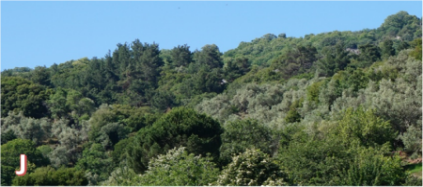

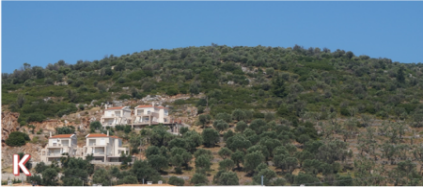

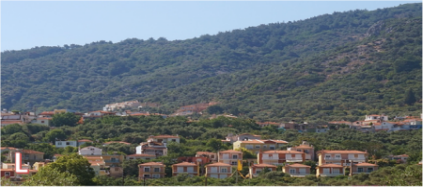

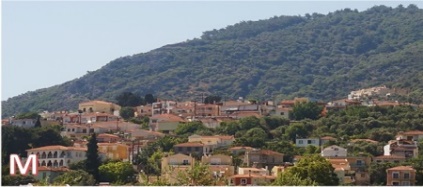

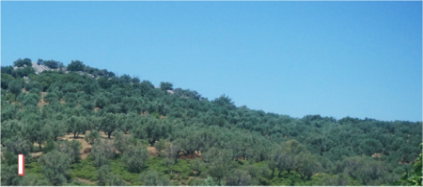


Mixed forest

Olive forest

Scattered housing

Sparsely built village/

Suburbs

Densely built village/

City


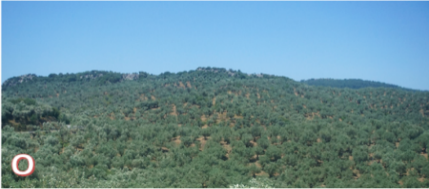

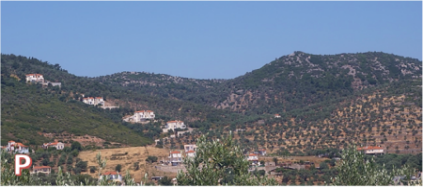

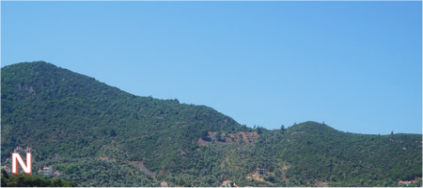

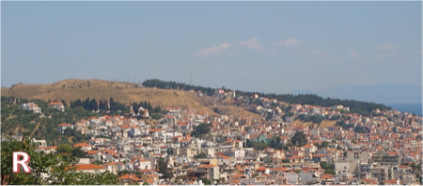

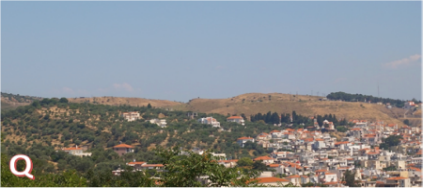


**J**

**I**

**Fig. 1** Landscapes ranked by tourists during the preference survey in respective sets illustrating processes of agricultural abandonment and housing sprawl (urbanization). Sets 1 and 2 utilize Google Street View (2015) panoramic imagery
